# Genome-Wide Analyses of Individual *Strongyloides stercoralis* (Nematoda: Rhabditoidea) Provide Insights into Population Structure and Reproductive Life Cycles

**DOI:** 10.1371/journal.pntd.0005253

**Published:** 2016-12-29

**Authors:** Taisei Kikuchi, Akina Hino, Teruhisa Tanaka, Myo Pa Pa Thet Hnin Htwe Aung, Tanzila Afrin, Eiji Nagayasu, Ryusei Tanaka, Miwa Higashiarakawa, Kyu Kyu Win, Tetsuo Hirata, Wah Win Htike, Jiro Fujita, Haruhiko Maruyama

**Affiliations:** 1 Division of Parasitology, Faculty of Medicine, University of Miyazaki, 9800 Kihara, Miyazaki, Japan; 2 Department of Environmental Parasitology, Tokyo Medical and Dental University, Yushima, Bunkyo-ku, Tokyo, Japan; 3 Department of Endoscopy, Ryukyu University Hospital. Okinawa, Japan; 4 Department of Infectious, Respiratory, and Digestive Medicine, Faculty of Medicine, University of the Ryukyus, 207 Uehara, Nishihara, Okinawa, Japan; 5 Department of Microbiology, University of Medicine 1, Yangon, Myanmar; University of Cambridge, UNITED KINGDOM

## Abstract

The helminth *Strongyloides stercoralis*, which is transmitted through soil, infects 30–100 million people worldwide. *S*. *stercoralis* reproduces sexually outside the host as well as asexually within the host, which causes a life-long infection. To understand the population structure and transmission patterns of this parasite, we re-sequenced the genomes of 33 individual *S*. *stercoralis* nematodes collected in Myanmar (prevalent region) and Japan (non-prevalent region). We utilised a method combining whole genome amplification and next-generation sequencing techniques to detect 298,202 variant positions (0.6% of the genome) compared with the reference genome. Phylogenetic analyses of SNP data revealed an unambiguous geographical separation and sub-populations that correlated with the host geographical origin, particularly for the Myanmar samples. The relatively higher heterozygosity in the genomes of the Japanese samples can possibly be explained by the independent evolution of two haplotypes of diploid genomes through asexual reproduction during the auto-infection cycle, suggesting that analysing heterozygosity is useful and necessary to infer infection history and geographical prevalence.

## Author Summary

*Strongyloides stercoralis*, one of the most neglected helminths causes strongyloidiasis mainly in tropical and subtropical regions worldwide. The parasite’s complex lifecycle includes sexual and asexual reproduction outside and inside the host, respectively. The parasite can also asexually complete a life cycle within the host's body, which is called autoinfection causing life-long infection. In order to investigate the population structure and transmission patterns of this parasite we sequenced individual nematodes isolated from human faeces in Japan and Myanmar, where the parasite is present at low and high frequencies, respectively. Whole genome sequencing of small parasites is generally difficult because the amount of DNA is limiting. However, we overcame this problem by combining whole genome amplification with next-generation sequencing. Sequence comparisons revealed 0.6% of the genome is variable among samples, and the variants showed clear separation by the location of their origin. We found that heterozygosity within the genomes was higher in Japan, which is likely explained by the predominance of asexual reproduction through auto-infection, suggesting that analyses of heterozygosity are required to better understand the history of a population.

## Introduction

The helminth *Strongyloides stercoralis*, which is one of the most common and globally distributed human pathogens of clinical importance, infects 30–100 million people worldwide [1,2]. This parasite most often resides in areas with tropical or subtropical climates and less frequently in areas with a temperate climate. It occurs infrequently in societies where faecal contamination of soil or water is rare, and therefore, new infections are very rare in countries with developed economies [3]. However, infection can persist for life unless effective treatment eliminates all adult parasites and migrating auto-infective larvae. Therefore, carriers are present in developed countries, representing a potential risk of horizontal transmission among humans [4]. *Strongyloides stercoralis* is also a natural parasite of dogs [5].

*Strongyloides stercoralis* is the only medically important nematode that can multiply in the host via an auto-infection cycle to reach critical levels and cause death [1,6,7]. The complex life cycle includes sexual and asexual reproduction. Infection with *S*. *stercoralis* begins when the infective third-stage larvae (iL3) in soil attach to and penetrate the human skin. After reaching the lung through the bloodstream, the parasites ascend to the trachea, and are swallowed to settle in the small intestine (their final destination) where the parasitic adults produce eggs through parthenogenesis. The larvae passed in the host faeces develop via either the homogonic route into iL3 forms or the heterogonic route into free-living adult stages that reproduce sexually outside the host. Although most eggs/larvae of the parasite are excreted from the host with faeces, homogonic larval development may occur inside the small intestine giving rise to auto-infective L3 which penetrate the intestinal wall and invade the tissues, ultimately entering the lung and returning to the small intestine to complete development to the parasitic female. In this circumstance, termed auto-infection, repeated generations of development may take place within a single host. [5]. Although strongyloidiasis is usually an indolent disease in immunocompetent hosts, it can cause a hyperinfective syndrome (disseminated strongyloidiasis) in immunocompromised hosts through the reproductive capacity of the parasite inside the host. Disseminated strongyloidiasis, if untreated, is associated with mortality rates of approximately 90% [8].

Despite its great medical importance, the threadworm *S*. *stercoralis*, is one of the most overlooked helminths [1]. The parasite's complex life cycle has long been considered a major impediment to attempts to control strongyloidiasis. Recently, the genome of *S*. *stercoralis* was sequenced and compared with other species of *Strongyloides* [9]. This comparative genomic study illuminates the use of genome-wide analysis to identify genes related to parasitism, to investigate diversity and population structures, and to determine the transmission route of *S*. *stercoralis*. Here, we aimed to determine the intra-species genomic variations of *S*. *stercoralis* present in Japan and Myanmar, which differ in socioeconomic status, history of infection and prevalence of this nematode.

## Methods

### Ethical statement

The Ethics Committees of the University of the Ryukyus and the University of Medicine-1 Yangon approved this study. Participants, who were informed of the study's aims and procedures, provided written informed consent. All individuals infected with *S*. *stercoralis* were treated with ivermectin.

### Sample collection

Faecal samples were collected in 2014 (Table 1) in Okinawa, Japan, representing an area where *S*. *stercoralis* is non-prevalent and where *S*. *stercoralis* has not been endemic for at least the last 50 years [10], and Htantabin, Myanmar as a prevalent area where new infections frequently occur. In Okinawa, Japan, faecal tests were performed for inpatients in one hospital and residents of two elderly nursing homes located in the southern part of Okinawa. For Myanmar samples, a community survey was conducted in three different villages of Htantabin area. Faeces were incubated on 2% (w/v) agar plates at 25°C for 2–4 days. This culture condition would allow a portion of parasites to undergo a complete free-living generation involving a sexual cross although worms may mate with their genetically identical siblings in the culture. Individual nematodes (iL3) that crawled out of the faeces were transferred to 0.2 ml tubes containing 10 μl of worm lysis solution (9 μl Direct PCR [Viagen], 0.5 μl of 20 mg/ml Proteinase K [Qiagen] and 0.5 μl of 1 M dithiothreitol [Wako]). The lysates were incubated at 60°C for 1 h and then at 95°C for 10 min. To identify nematodes, the 18S ribosomal RNA gene was amplified using 0.1 μl of worm lysate with the primers 988F and 1912R [11], and the amplicons were sequenced using an ABI 3130 sequencer (Applied Biosystems) with the BigDye Terminator v3.1 kit. Worm lysates were immediately used for further analysis or stored at −30°C.

**Table 1:** *Strongyloides stercoralis* samples used in this study.

Sample ID	Host ID	Host gender	Host age	Collection site	Collection country	Collection date
MyHTB10-5	MyHTB10	M	58	Village A, Htantabin	Myanmar	August, 2014
MyHTB10-6						
MyHTB10-7						
MyHTB122-2	MyHTB122	M	33	Village B, Htantabin		
MyHTB122-6						
MyHTB122-8						
MyHTB177-4	MyHTB177	M	38	Village C, Htantabin		
MyHTB177-5						
MyHTB177-6						
Rk4-1	Rk4	F	65	Hospital A, south Okinawa	Japan	January, 2014
Rk4-6						
Rk4-7						
Rk4-8						
Rk4-29						
Rk4-30						
Rk5-6	Rk5	M	58	Hospital A, south Okinawa		
Rk5-10						
Rk5-12						
Rk5-14						
Rk6-1	Rk6	F	104	Nursing home B, south Okinawa		February, 2014
Rk6-2						
Rk6-3						
Rk6-4						
Rk7-1	Rk7	F	79	Hospital A, south Okinawa		
Rk7-2						
Rk7-4						
Rk7-5						
Rk8-3	Rk8	F	91	Hospital A, south Okinawa		
Rk8-7						
Rk8-8						
Rk9-3	Rk9	M	70	Nursing home C, south Okinawa		
Rk9-6						
Rk9-11						

### Whole genome amplification (WGA)

Genomic DNA was amplified from 1 μl of worm lysate using an Illustra GenomiPhi V2 kit (GE Healthcare) according to the manufacturer’s protocol. Amplified products were quality-checked using 1% agarose gel electrophoresis, purified using a QIAamp DNA Mini Kit (Qiagen) and quantified using Qubit (Life Technologies).

### Illumina sequencing

Libraries were constructed using a Nextera DNA Sample Prep Kit (Illumina) with 100 ng of amplified DNA according to the manufacturer’s protocol. The libraries were sequenced using an Illumina MiSeq with a v3 Reagent kit (600 cycles) according to the manufacturer’s recommended protocol (https://icom.illumina.com/) to produce 300-bp paired-end reads to obtain ~3G base data. Non-WGA reads of the genome reference strain (SSTP) were obtained from NCBI SRA under accession number ERR066168, randomly sampled and used as a reference to evaluate WGA reads.

### Variant calls

We used Trimmomatic [12] to eliminate adapter contamination from the reads and achieve a minimum quality score = 15 (SLIDINGWINDOW:4:15) before mapping against the *S*. *stercoralis* reference genome (ver. 2.0.4) [9] using SMALT v0.7.4 (https://www.sanger.ac.uk/resources/software/smalt/) with options–x (each mate is mapped independently) and–y 0.8 (mapping to the region of highest similarity in the reference genome at a similarity threshold > 80%). Duplicate reads were marked using the Picard tool (ver. 1.95), and indels were realigned with GATK (version ver. 3.3.0) [13] using the IndelRealigner. Variants were then called using GATK HaplotypeCaller. Variants were annotated using GATK and ANOVA (ver. 2014-11-12). Depth of coverage was calculated by counting mapped reads per site using GATK DepthOfCoverage [13]. Analysis of population genetics, including calculating nucleotide diversity (π) and inbreeding coefficient (F_IN_), were performed using vcftools (v0.1.12b) [14]. Mean of per-site nucleotide diversities between two genomes were reported as a pair-wise genome distance. Analysis of molecular variance (AMOVA) was conducted with R Poppr package [15]. Other statistical analyses were performed using R (ver 3.1.1) and in-house python scripts. In the previous study, using *C*. *elegans* as a model, we found WGA variant calls with low coverage data tends to call heterozygous loci homozygous [16]. To avoid this bias toward calling homozygous sites, we excluded relatively low coverage samples comprising < 70% of genomic regions with 15× depth (nematodes designated MyHTB122-6, Rk5-6, Rk6-4, Rk7-5, Rk8-3 and Rk8-8) from the heterozygosity-related analyses.

### Principal component analysis

Principal component analysis (PCA) was performed using R (ver 3.1.1) implemented with SNPRelate package [17]. Bi-allelic SNPs were extracted from full variant information of all the samples and used for PCA analyses.

### Reconstruction of mitochondrial genomes

The mitochondrial genomes of Rk4-1 nematodes were reconstructed from the Illumina reads using MITObim ver 1.6 [18]. In the first step, Illumina reads were mapped to the *S*. *stercoralis* reference sequence (Genbank accession No. NC_028624) to generate a seed for the second step. In the second step, gaps and ambiguous regions in the seed were replaced by iterative mapping that was repeated until all gaps were closed, and the number of reads remained constant. Reconstructed mitochondrial sequences were refined by correcting bases using ICORN2 [19], and the assembly was used to represent the Japanese nematode reference mitochondrial genome.

### Phylogenetic reconstruction

Nucleotide sequences of SNP positions in scaffolds > 30 kb, which accounted for 96% of the total genome assembly, were extracted from the vcf files and were used to construct phylogenetic networks based on similarity/dissimilarity with the Neighbor Net method of SplitsTree4 [20]. Computational phasing of the diploid genotypic data was performed using SHAPEIT2 with its default parameters [21]. Phased sequence data from all samples were used to create a separate Maximum Likelihood tree using FastTree (ver 2.1.8) for each scaffold > 30 kb [22].

To generate a mitochondrial-based phylogeny, reads from each nematode sample were mapped to the Japanese parasite's reference sequence (see above) using SMALT v0.7.4, and SNPs were called using GATK [13]. The nucleotide sequences of the SNPs were extracted and used to generate Maximum Likelihood trees using FastTree (ver 2.1.8) [22].

### Accession numbers

All sequence data were submitted to the DDBJ Sequence Read Archive (DRA) under project accession number PRJDB5112.

## Results

### Whole genome amplification and re-sequencing

We re-sequenced the genomes of 33 *S*. *stercoralis* nematodes collected in Myanmar (prevalent region, nine from three patients) and Japan (non-prevalent region, 24 from six patients) [10] (Table 1). We applied the WGA method [16] using the Illumina MiSeq to sequence the whole genome of a single nematode. We obtained 300-bp paired-end reads to > 20× coverage (> 3 Gb) for each nematode and mapped them to the *S*. *stercoralis* reference genome. The mapping ratios of each sample to the reference genome ranged from 77.46% to 96.96%, and the ratios for reads mapped in the correct orientation and distance (‘proper paired’ reads) ranged from 48.94% to 62.72% (S1 Table). In contrast, the mapping ratios of non-WGA reference reads were 90.95% with 71.79% proper pairs (S1 Table, S1 Fig). Although amplification bias depending on genome locations were observed in the WGA samples (S2 Fig), > 10× coverage was achieved for > 80% of the genomic locations, and the median coverage values ranged from 20 to 50 for most samples (S1 Table, S1 Fig).

### Variant calls

We detected 298,202 variant positions, which accounted for 0.6% of the total genome, among the 33 samples when compared with the reference. Most variants were SNPs (231,583 positions), and small inserts or deletions (indels) were present at 67,655 positions (S2 Table). The number of variant positions in individual nematodes (including homozygous and heterozygous sites compared with the reference) ranged from 137,439–146,259 and 135,583–157,900 of the Myanmar and Japanese samples, respectively (S2 Table).

**Fig 1:**
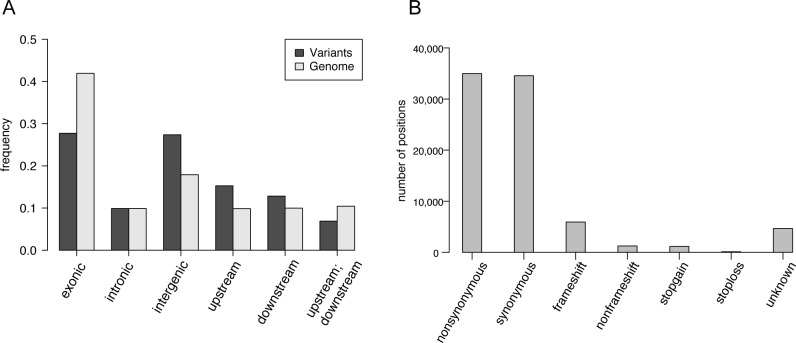
Variant position percentage/numbers across sequence classes. (A) Intergenic—variant resides in the intergenic region, not included in upstream or downstream regions. Intronic—variant overlaps an intron. Exonic—variant overlaps a coding region. Upstream—variant overlaps a 1-kb region upstream of the transcription start site. Downstream—variant overlaps a 1-kb region downstream of the transcription termination site. Upstream;downstream—variant is located in both downstream and upstream region (possibly for 2 different genes). Genome percentages of the same classes are shown alongside. (B) Effects of the exonic variants. Nonsynonymous—a single nucleotide change that changes an amino acid residue; Synonymous—a single nucleotide change that does not change an amino acid residue; Frameshift—an insertion or deletion of one or more nucleotides that cause a frameshift; Stop gain/loss—a nonsynonymous SNP or indel that creates or eliminates a stop codon at the variant site; Unknown—unknown function (caused by errors in the gene-structure definition in the database).

Comparisons with reference gene models revealed that 27.7% of the variants were located in intergenic regions, followed by 27.3%, 15.2%, 12.8% and 9.9% in exonic, upstream, downstream and intronic regions, respectively (Fig 1A). There were higher frequencies of variant positions in intergenic regions compared with those of the individual nucleotides in the total genome and lower frequencies of variant positions in exonic regions (Fig 1A).

In the exon variations, similar numbers of synonymous and non-synonymous SNPs were detected in 34,551 and 34,960 positions, respectively (Fig 1B). Frameshift indels and stop mutations were less frequent (5,932, 1,237, 1,158 and 119 for frameshift, non-frameshift, stop-gain and stop-loss, respectively) (Fig 1B).

The distribution of SNPs along the four longest scaffolds is shown in S3A Fig and the distribution of numbers of SNPs by 10-kb window for scaffolds bigger than 100 kb are shown in S3B Fig. Variants were unevenly distributed along the genome with numbers of variant positions in 10-kb window ranging from 3 to 922 (median = 31), suggesting that they represented ‘hotspots’. Further, the hotspot regions did not correspond to regions of high coverage mapping (S2 Fig and S3 Fig) (Pearson’s r = -0.01), suggesting that the variant call was not significantly influenced by WGA amplification bias. No significant differences in SNP distribution between the two countries were observed (high correlation coefficient between SNP numbers in 10-kb window of the two countries; Pearson’s r = 0.78, p < 2.2e-16).

### Population structure

**Fig 2:**
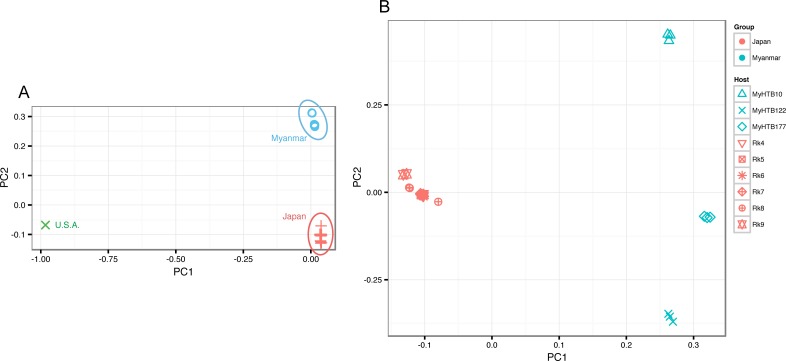
Principal component analyses of variant data. (A) a plot including the reference genome strain (USA). Variances represented by PC1 = 40.1% and PC2 = 14.1%. (B) Japanese and Myanmar samples only. Variance represented by PC1 = 28.4% and PC2 = 10.3%. Totals of 234,398 and 128,040 variant positions were included in the PCA analysis for Fig 2A and 2B, respectively.

Principal component analysis (PCA) of SNPs compared with the reference strain unambiguously separated the Japanese and Myanmar samples from the reference strain by the first PC, which account for 40.1% of the variance. Japanese and Myanmar samples were separated by the second PC (14.1% of variance) (Fig 2A). Fig 2B shows the PCA results without the reference. The Myanmar and Japanese samples were separated by PC1 (28.4%). PC2 (10.3%) grouped the Myanmar samples according to their host origins, although the separation in the Japanese samples was not unambiguous.

**Fig 3:**
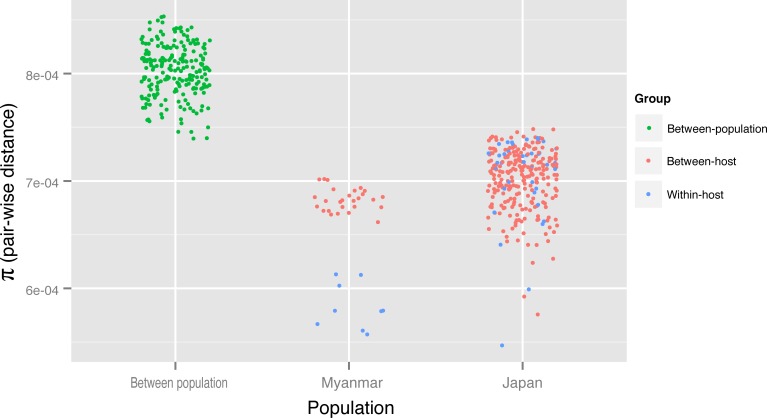
Pair-wise genetic distances (π) of genomes between samples. Distances between samples from Japan and Myanmar (green dots) were generally higher than those of the same country. Inter-host comparisons (red dots) show higher πvalues than those of within-host comparisons (blue dots) in Myanmar. The differences are ambiguous in the Japanese samples.

Pair-wise distances (π) of samples originated from different countries (Japan vs. Myanmar) were generally higher than those within populations (Fig 3). In the Myanmar samples, pair-wise distances between hosts were higher compared to those within hosts, although such differences were not observed in the Japanese samples (Fig 3). Because the parasitic adult stage of *Strongyloides* is mitotically parthenogenetic, multiple larval progeny of such adults will be, in theory, genetically identical. Although within-host samples showed high similarity to each other (π values < 7.5e-04) both in Japan and Myanmar, they still exhibited some differences from each other. Because of possibility of errors in WGA or sequencing process and difficulty in heterozygous SNP call, it is difficult to conclude that they are genetically different or identical progeny. Simulated experiments using proved progeny of single adults will be useful to answer this question.

Analysis of molecular variance (AMOVA) showed 23.7% of variance was associated with differences between populations and 6.3% with differences between hosts, whereas more than 100% of the variance was attributed to variation within samples (S3 Table). Although the negative phi-statistics and variance values observed in AMOVA (S3 Table) may reflect problems with sample size and analytical strategy, these results suggest a close relationship among the Japanese samples independent of host origin and high heterozygosity within the individual genomes.

**Fig 4:**
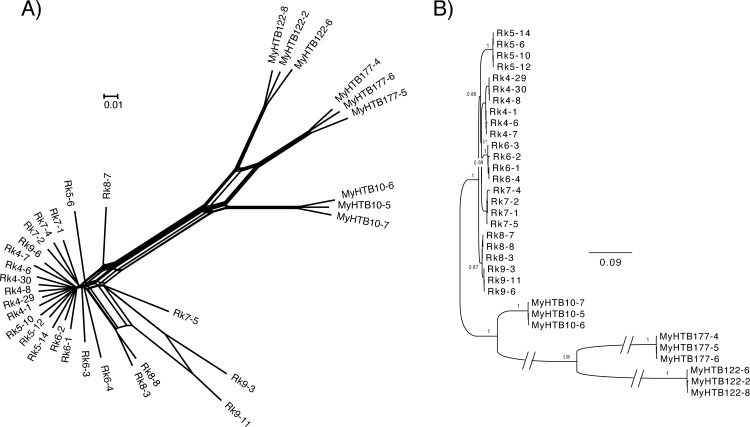
(A) Phylogenetic network analyses based on SNPs in the genome of *S*. *stercoralis*. (B) A maximum likelihood tree of SNPs in the mitochondrial genomes of *S*. *stercoralis* samples. The scale bars show the number of nucleotide substitutions per site. Branches marked with \\ indicate a two-fold shortening of branches (only for practical purposes).

Next, we constructed phylogenetic networks according to the SNPs, which support the PCA results (Fig 4). The tree contained two main clades, comprising Myanmar or Japanese samples. All samples in the Myanmar clade from the same host clustered together and were clearly distinct from those of other hosts. Most Japanese samples sub-clustered according to host origin, although the separations were not as clear as those of the Myanmar samples. Further, we found some Japanese samples (Rk5-6, Rk7-5, Rk8-3 and Rk8-8), which have lower coverage (S1 Table), were placed at positions distant from those of other worms of the same host origin. This is likely because of failure to call heterozygous SNP in low coverage samples [16]. We therefore removed these four samples (Rk5-6, Rk7-5, Rk8-3 and Rk8-8) and those having lower coverage than the four samples (based on % of genome regions with 15× coverage; MyHTB122-6 and Rk6-4) from further analyses. Two samples from host Rk9 (Rk9-3 and Rk9-11), which had higher coverage (S1 Table), occupied positions more distant from the other samples as well as a sample from host Rk9 (Rk9-6) (Fig 4A).

Next, we used the computationally-phased sequence dataset for the Japanese samples to construct phylogenetic trees for each scaffold (> 100 kb). The two haplotypes in a genome, shown as A and B haplotypes in S4A Fig, separated into distinct clusters for most of samples. This result suggests that the haplotypes in the diploid genomes of most samples evolved independently. Haplotypes of samples Rk8-7, Rk9-3 and Rk9-11 exhibited distinct haplotype organisations in each-scaffold tree (shown in black colour in S4A Fig). In Myanmar samples segregation of two haplotypes was not clear compared to the Japanese samples and individual scaffold trees showed various patterns (S4B Fig), suggesting past occurrences of chromosome exchange and/or recombination between Myanmar samples.

The mitochondrial tree exhibited a similar topology to the nuclear tree (Fig 4B). The Japanese samples were placed into one clade, clearly separated from Myanmar samples with a high support value. Within the Japanese samples, those from host Rk9 clustered with those from host Rk8 and occupied the basal position of the other Japanese samples. As observed in the phylogenies of nuclear genomes, the samples from hosts Rk4, Rk5, Rk6 and Rk7 were closely related, but were unambiguously sub-grouped according to host origin. Samples from host Rk9 (Rk9-3, Rk9-6 and Rk9-11), which clustered separately in the nuclear tree, grouped together in the mitochondrial tree (Fig 4B). Interestingly, mitochondrial genome sequences of worms from the same host origin were not perfectly identical (especially in worms from host Rk4) although differences were very small and this may be due to sequencing errors.

### Genomic heterogeneity

*Strongyloides stercoralis* employs distinct modes of reproduction as follows: asexual parthenogenetic reproduction by parasitic females inside the host and sexual reproduction by free-living adults outside the host. Asexual reproduction may promote increased heterozygosity because of the absence of recombination and segregation in diploids (known as Mullers’s ratchet or Meselson effect) [23,24]. We therefore compared the heterozygosities (π_t_) of samples from Japan, where the parasites likely persist longer in the host through asexual auto-infection because no new infections are suggested to be unlikely to have occurred in Japan in the last 50 years [10] and our Japanese samples were collected from elderly people (Table 1), and samples from Myanmar to represent frequent new infections by larvae that arose through sexual reproduction.

As expected, most Japanese samples (i.e. all except Rk9-11, Rk9-3 and Rk8-7) comprised higher heterozygosities (π_t_ = 0.0015–0.0017 in scaffolds > 8 kb) compared with Myanmar samples (0.0011–0.0013) (S2 Table), and this difference was significant (P < 1.8e -5, Welch t-test, df = 23). Intra-genome heterozygosity does not seem to be highly associated with read depth (S5 Fig), and the excess of heterozygosity in the Japanese samples were consistently observed in the genome (S6 Fig). These results suggest that excess of heterozygosity in the Japanese samples is likely to be true, excluding the possibility of false calls due to contaminations or other uncertain factors. The negative inbreeding coefficients (F_IN_) observed in such Japanese samples (−0.36 to −0.22) may represent repeated parthenogenetic reproduction of the nematodes in their hosts (S2 Table). Exceptions were Rk9-11, Rk9-3 and Rk8-7, which comprised fewer heterozygosities (0.0009 to0.0013) and higher F_IN_ values (−0.09 to 0.28). The Japanese samples deviated significantly from Hardy-Weinberg equilibrium at 34.4% of loci, with 99.3% in heterozygous excess, compared with 0.8% of the loci in Myanmar samples, none of which were in heterozygous excess (S4 Table), suggesting more frequent asexual reproduction (insufficient sexual reproduction) has been used by Japanese worms than Myanmar ones. This point was discussed in [25] with observation of deviation from Hardy-Weinberg equilibrium in some populations of rat *Strongyloides* (*S*. *ratti*) and also reviewed in [26].

**Fig 5:**
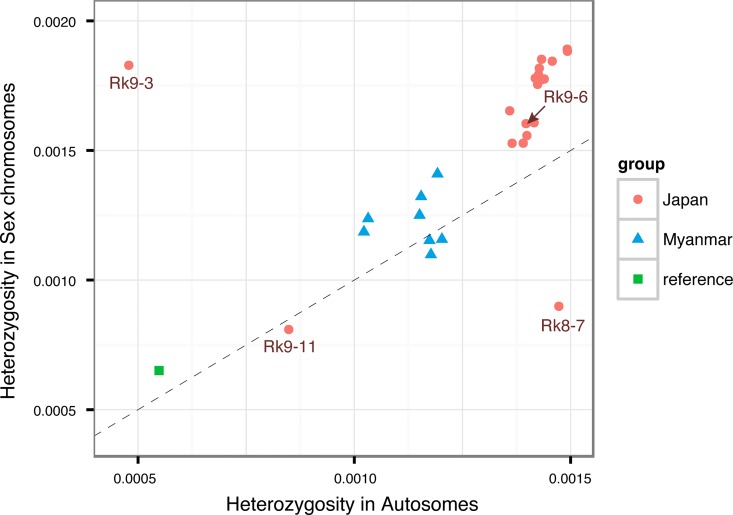
Intra-genomic heterozygosity in autosomes and sex chromosomes. Frequencies of heterozygosity (π_t_) per nucleotide site for the autosomal and sex chromosomal scaffolds are plotted in x and y axis, respectively. Three areas were shown by different colours and shapes. Broken line represents Y = X.

Next, we compared the heterozygosities of the scaffolds assigned to autosomes and sex chromosome [9] of individual samples (Fig 5). Two main groups were observed as follows: 1. Myanmar samples with values ranging from 0.001–0.0015 in the sex and autosomal scaffolds, 2. The majority of Japanese samples had with higher heterozygosities compared with those of Myanmar samples in the sex and autosomal scaffolds. The exceptions Rk9-11, Rk9-3 and Rk8-7 were positioned separately from those shown in the plot. The autosomal heterozygosities of Rk9-3 were lower but had values similar to those of the sex chromosomes of the other Japanese samples, whereas the heterozygosities of the sex chromosomes of Rk8-7 were low and had a value consistent with that of the autosomes of the Japanese samples. The values of both the sex chromosomes and autosomes of Rk9-11 were low. In contrast, the numbers of homozygous SNP sites in these three samples (S7 Fig) were greater than other Japanese samples on the sex chromosome of Rk8-7, the autosomes of Rk9-3 and both types of chromosomes of Rk9-11 (with an increase of approximately 50% of autosomes compared with Rk9-3).

Together, these results suggest that samples Rk8-7, Rk9-3 and Rk9-11 arose through recent sexual crossing between very closely-related individuals and acquired more homozygous chromosome pairs in sex chromosomes and autosomes. These findings likely explain the positions of Rk9-3 and Rk9-11 in the network tree, which were distant from Rk9-6 (Fig 4).

## Discussion

A major weakness of research on parasitic helminth genomes is the inability to obtain sufficient quantities of DNA because at present, none of these parasites can be cultured through its entire life cycle outside of a living host. Nevertheless, the WGA technique may solve this problem by producing high yields of whole genomic DNA from a single parasite [16]. Here we used the WGA technique combined with the NGS technology to re-sequence the entire genomes of individual *S*. *stercoralis* to acquire a better understanding the population structure of this medically important human pathogen. To the best of our knowledge, this study represents the first genome-wide approach to estimate the genotypic variations in *S*. *stercoralis* populations.

We show here that WGA detects variants with sensitivity comparable with those of normal variant detection methods, although WGA requires more data (coverage) to correctly call heterozygous positions, likely because of amplification bias. Here, our analysis of nematodes collected in Japan and Myanmar detected approximately 0.3 million variant positions, representing 0.6% of the genome, by comparison to the reference strain isolated from a dog in the United States. Although the reference and samples in this study were originally isolated from different hosts, this level of diversity represents as low as the diversity of *C*. *elegans* (~0.05%) [27] compared with other nematodes such as *Pristionchus pacificus* (~2%) [28] and *Bursaphelenchus xylophilus* (~4%) [29]. This may be explained by the relatively recent divergence of *S*. *stercoralis* from a common ancestor of *S*. *stercolaris* and the sister species, stronger selective pressure on the obligate parasite compared with free-living organisms such as *P*. *pacificus*, or facultative parasites such as *B*. *xylophilus* or both. Additionally, the unique mode of reproduction of this species may have affected the diversity level. *S*. *stercoralis* is distributed worldwide in areas with warm climates, and it will be interesting to analyse the diversity of *S*. *stercoralis* isolated in Africa, South America and Australia to study their global diversity. The data from such an analysis may illuminate the origin and migration routes of *S*. *stercoralis* and allow comparison of these attributes in populations of the parasite in humans and dogs as gene flow of parasites are generally determined by host movement [30].

Besides the human strongyloidiasis situations in the two countries (Japan and Myanmar), situations of *Strongyloides* infection in dogs are also likely to differ between the two countries. *Strongyloides* infection rate in dogs was reported to be as low as 0.4% in Okinawa, Japan [31]. Although we can’t find any reports about Myanmar canine strongyloidiasis, infection rate in Myanmar is possibly very high as reported in other Southeast Asian countries [32,33]. Therefore, a genome-wide investigation of their population structures would be of interest to see if a similar intra-genome heterozygosity trend can be observed as in human *Strongyloides* and to identify if there are interspecies transmissions between dogs and humans.

The phylogenetic relationships inferred from nuclear and mitochondrial SNPs were basically similar to each other. However, the relationships of Japanese samples observed in the nuclear trees were more complicated and therefore difficult to interpret. We found this is likely not only because the Japanese samples originated from a small gene pool but is also potentially explained by independent evolution of two haplotypes of the diploid genomes through asexual reproduction. This suggests that analyses of heterozygosity (e.g. by phasing) are useful and necessary to gain a better understanding of the structures of populations of *S*. *stercoralis*.

Because *S*. *stercoralis* has not been endemic in Japan for decades [10], the Japanese samples collected from elderly hosts aged 58–104 years (Table 1) may have been maintained only by auto-infection cycles for a long time. The higher heterozygosity of Japanese compared with Myanmar samples is thus possibly explained by an accumulation of heterozygous positions during the auto-infection cycle [10]. The exceptions Rk9-3, Rk9-11 and Rk8-7, which have reduced heterozygosity in sex or autosomal scaffolds or both are likely explained by recent cross events between two very closely related individuals, possibly during their isolation from a faecal culture. This, in turn, provides robust evidence that parthenogenesis of the parasitic female is mitotic (non-meiotic) and that free-living adults exchange chromosomes outside the host. Further, positive F_IN_ (inbreeding efficiency) values of the Myanmar samples suggest that new infections occur in the prevalent regions by infective larvae produced through sexual reproduction between closely related individuals.

It has been suggested that new infections are unlikely to have occurred in Japan in the last 50 years [10]. Assuming that the genomic mutation rate of *S*. *stercoralis* is the same as that of *C*. *elegans* (9*10^−9^/site/generation) [34] and the minimum *S*. *stercoralis* generation time is 8 days, 50 years of asexually cycling within a human host can cause approximately 1,900 heterozygous sites to accumulate in the 86-M base diploid genome. Although this value is high, it is only ~20% of the number of differences observed between samples isolated in Japan and Myanmar (S2 Table). These values suggest that the frequency of sexual reproduction, which can reduce heterozygosity, is also an important factor for determining the number of heterozygous sites in the nematode genome. The analysis of heterozygosity can therefore serve to help draw inferences about the history of infections and the prevalence of parasites in a specific area.

## Supporting Information

S1 Table Mapping statistics of 300-base paired-end reads to the reference genome. Table shows number and ratio of reads mapped to the S. stercoralis reference genome, ratio of reads mapped in pairs in proper directions and distance, sum of mapped bases, mean and median coverage attained and ratio of genome positions that exceed coverage of 10× and 15×.
(XLSX)

S2 Table Variant statistics for individual nematode samples. Table shows number of variant positions, SNPs and Indels, number of transitions and transversions within SNP changes and the two ratio, number and ratio of heterozygotic SNPs in scaffolds bigger than 8 kb, and inbreeding coefficient (F_IN_) estimated on per-individual basis.
(XLSX)

S3 Table Analysis of molecular variance (AMOVA) for 10,000 randomly selected SNPs of *S*. *stercoralis* samples in Japan and Myanmar. (XLSX)

S4 Table Testing Hardy-Weinberg equilibrium across the *S*. *stercoralis* genome. (XLSX)

S1 Fig Distribution of median depth of coverage by 5 kb windows along the reference genome. SSTP; non-WGA reference strain. The boxes indicate median, 25^th^ and 75^th^ percentile. Whiskers extend to the minimum and maximum values, which are no more than 1.5 times the interquartile range from the box, while outliers are shown by dots.
(PDF)

S2 Fig Mapping depth of coverage (number of reads) of WGA samples MyHTB177-4, Rk4-29, Rk6-2 and the non-WGA reference strain in the biggest four scaffolds. Normalised coverage in 5kb-window (the absolute coverage divided by the median coverage of all the genome sites) was shown in y-axis.
(PDF)

S3 Fig A) Number of variant positions among 33 studied samples in 10-kb window along the four largest scaffolds/contigs in Japanese and Myanmar *S*. *stercoralis* compared with the reference genome. B) A histogram showing SNP number distributions in 10-kb window for scaffolds larger than 100 kb.
(PDF)

S4 Fig Maximum likelihood trees of phased haplotype sequences of Scaffold000001, Scaffold000002, Scaffold000003, Scaffold000004, Contig000005, Scaffold000006 (autosomes) and Scaffold000007, Scaffold0000010, Scaffold0000011 (sex chromosomes). Trees were constructed using FastTree and visualised using FigTree. Two haplotypes from a diploid genome are coloured in either red or blue.
(PDF)

S5 Fig Comparison of depth of coverage of the whole genome sites and of heterozygous SNP sites, suggesting heterozygous SNP call is not highly affected by depth of reads coverage. (PDF)

S6 Fig Distribution of heterozygous SNP sites in Japanese and Myanmar samples along in 10-kb window along the four largest scaffolds/contigs. Excess of heterozygosity sites in Japanese samples are consistently observed in the scaffolds. Out of 1175 windows, 941 (80.1%) and 205 (17.4%) show excess in Japanese and Myanmar samples, respectively and the difference between the two proportion is significant (Z test; p< 2.6e-174).
(PDF)

S7 Fig Frequencies of homozygous SNPs per nucleotide position in autosomes and sex chromosomes. Three areas were shown by different colours and shapes. Broken line represents Y = X.
(PDF)
