# Supplementary material for: Genome-Wide Analyses of Individual Strongyloides stercoralis (Nematoda: Rhabditoidea) Provide Insights into Population Structure and Reproductive Life Cycles
Source: PLoS Negl Trop Dis. 2016 Dec 29;10(12):e0005253. doi: 10.1371/journal.pntd.0005253 (PMC5226825; doi:10.1371/journal.pntd.0005253)
Supplement: S1 Fig — SSTP; non-WGA reference strain. The boxes indicate median, 25th and 75th percentile. Whiskers extend to the minimum and maximum values, which are no more than 1.5 times the interquartile range from the box, while outliers are shown by dots. (PDF) [file pntd.0005253.s005.pdf]

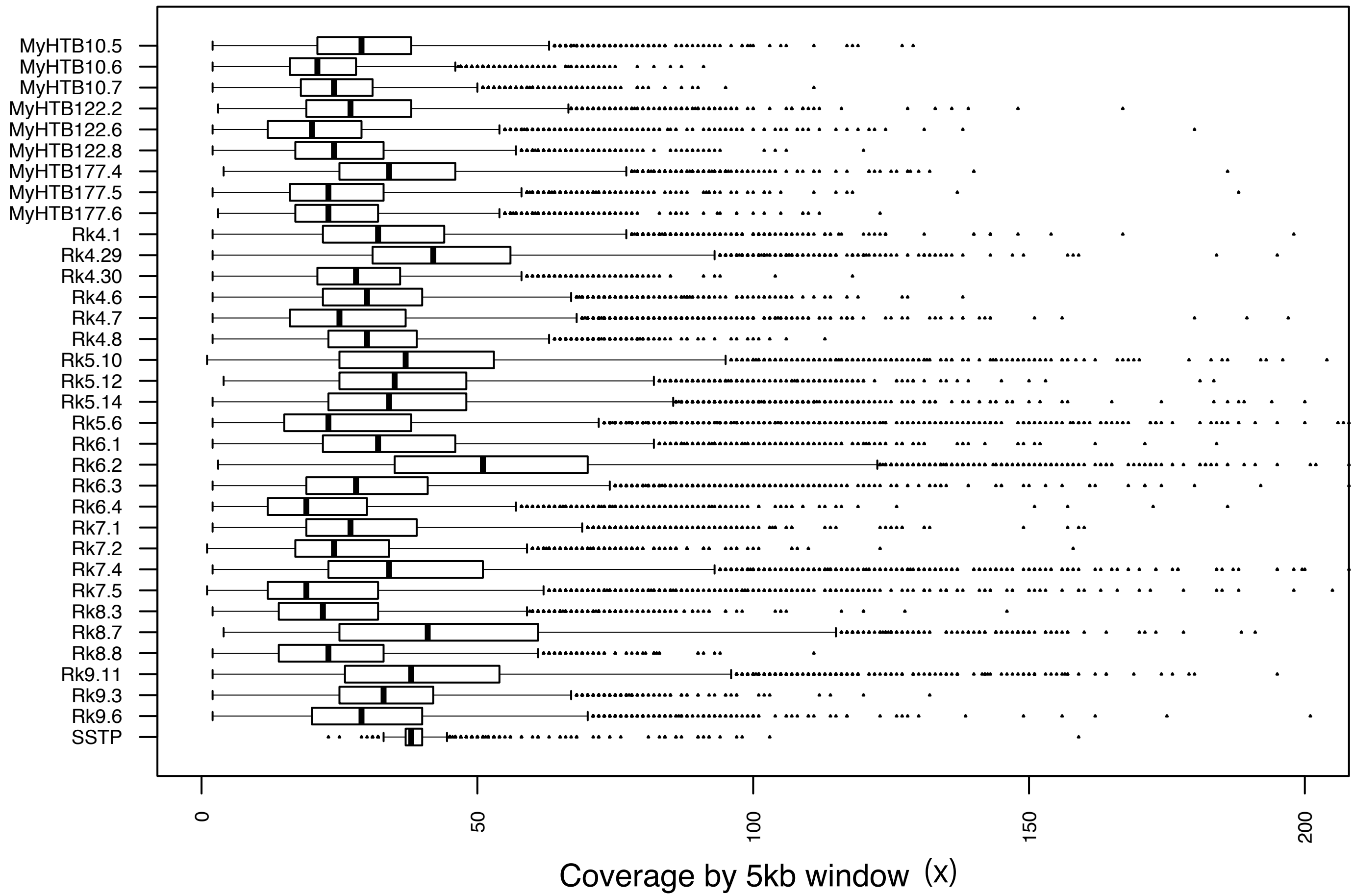

S1 Fig. Distribution of median depth of coverage in 5-kb windows along the reference genome. SSTP; non-WGA reference strain. The boxes indicate median, 25th and 75th percentile. Whiskers extend to the minimum and maximum values which are no more than 1.5 times the interquartile range from the box, while outliers are shown by dots.
