# Supplementary material for: Genome-Wide Analyses of Individual Strongyloides stercoralis (Nematoda: Rhabditoidea) Provide Insights into Population Structure and Reproductive Life Cycles
Source: PLoS Negl Trop Dis. 2016 Dec 29;10(12):e0005253. doi: 10.1371/journal.pntd.0005253 (PMC5226825; doi:10.1371/journal.pntd.0005253)
Supplement: S2 Fig — Normalised coverage in 5kb-window (the absolute coverage divided by the median coverage of all the genome sites) was shown in y-axis. (PDF) [file pntd.0005253.s006.pdf]

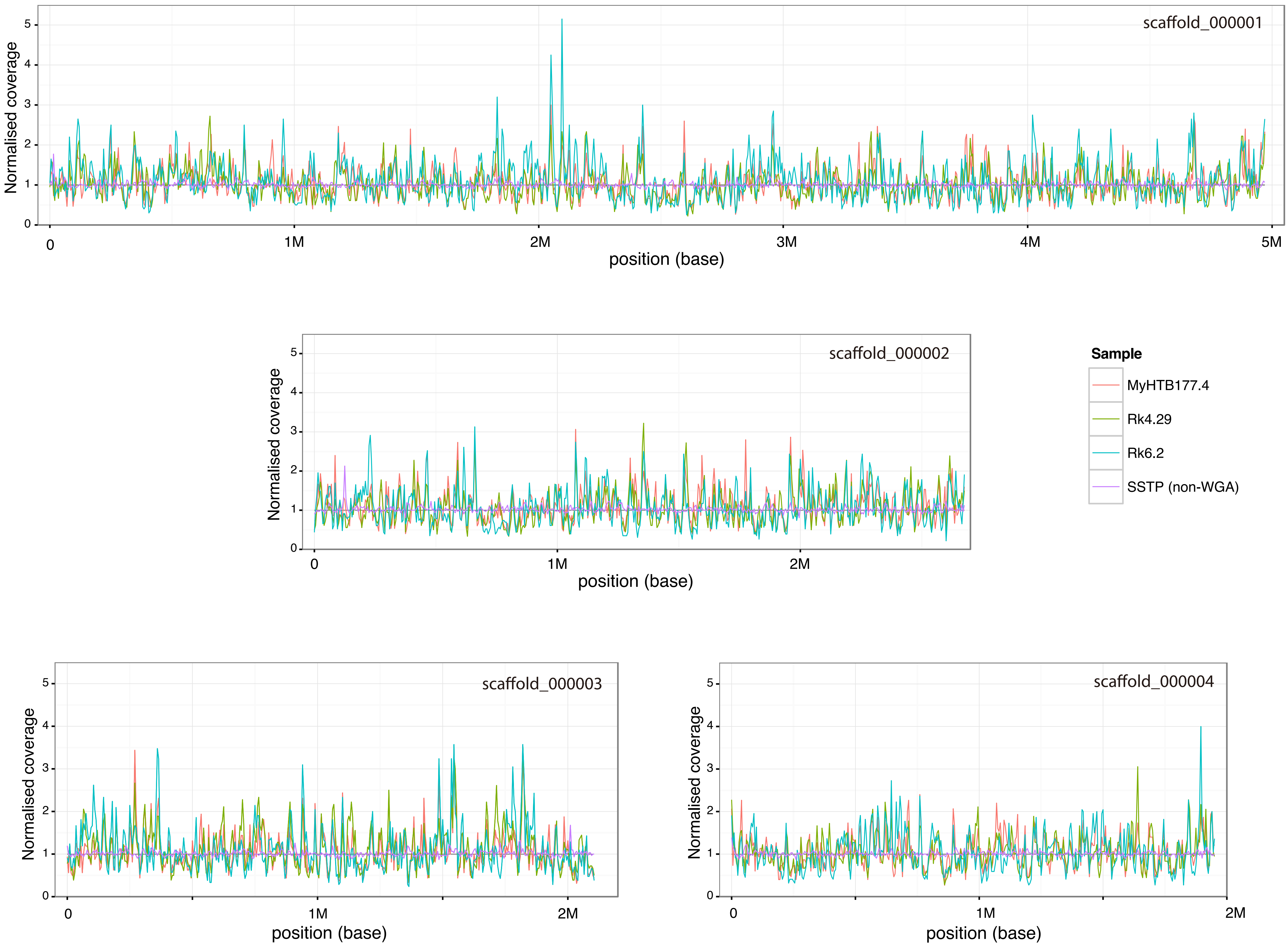

S2 Fig. Mapping depth of coverage (number of reads) of WGA samples MyHTB177-4, Rk4-29, Rk6-2 and the non-WGA reference strain in the biggest four scaffolds. Normalised coverage in 5kb-window (the absolute coverage divided by the median coverage of all the genome sites) was shown in y-axis.
