# Supplementary material for: Genome-Wide Analyses of Individual Strongyloides stercoralis (Nematoda: Rhabditoidea) Provide Insights into Population Structure and Reproductive Life Cycles
Source: PLoS Negl Trop Dis. 2016 Dec 29;10(12):e0005253. doi: 10.1371/journal.pntd.0005253 (PMC5226825; doi:10.1371/journal.pntd.0005253)
Supplement: S3 Fig — A) Number of variant positions among 33 studied samples in 10-kb window along the four largest scaffolds/contigs in Japanese and Myanmar S. stercoralis compared with the reference genome. B) A histogram showing SNP number distributions in 10-kb window for scaffolds larger than 100 kb. (PDF) [file pntd.0005253.s007.pdf]

A)

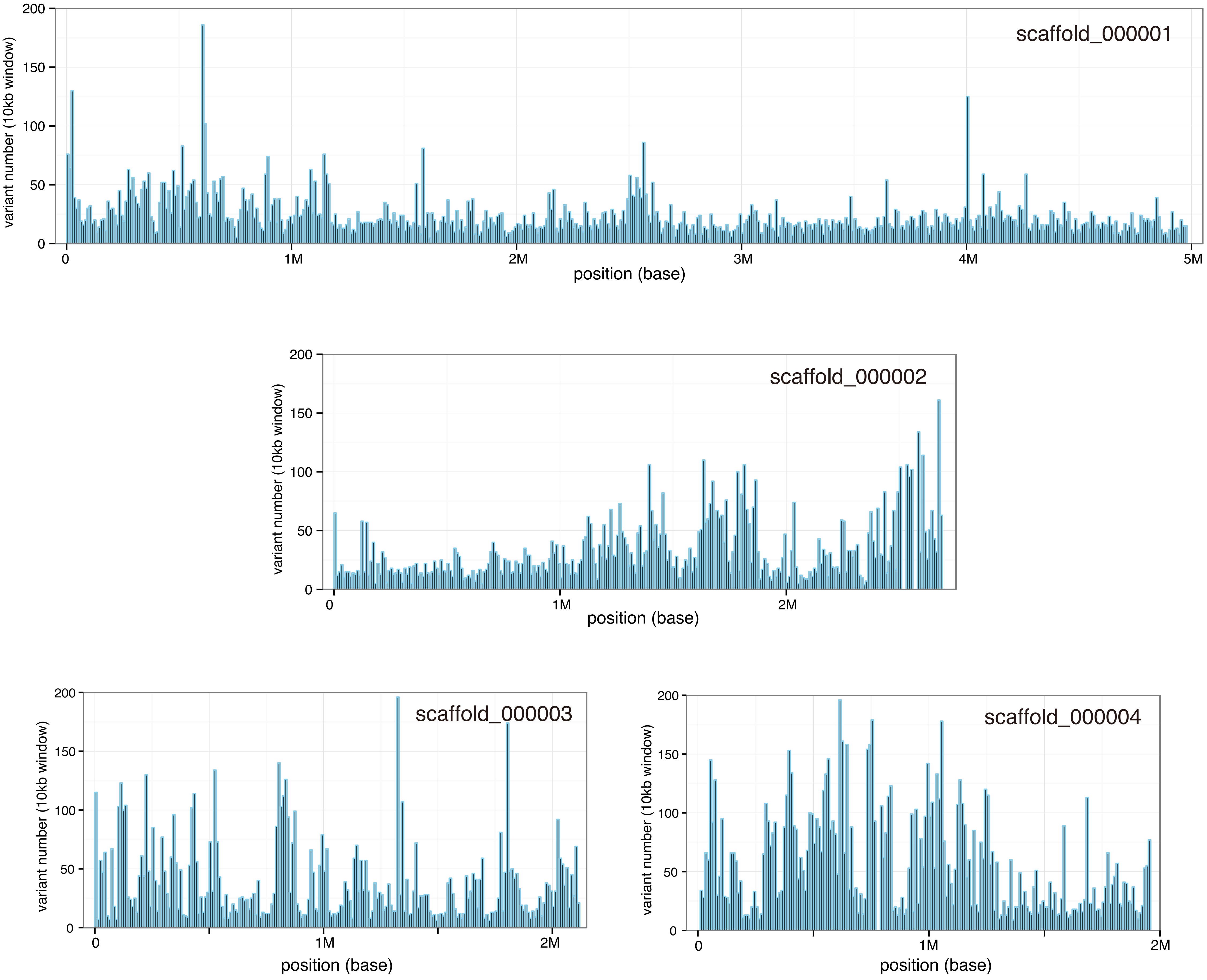

B)

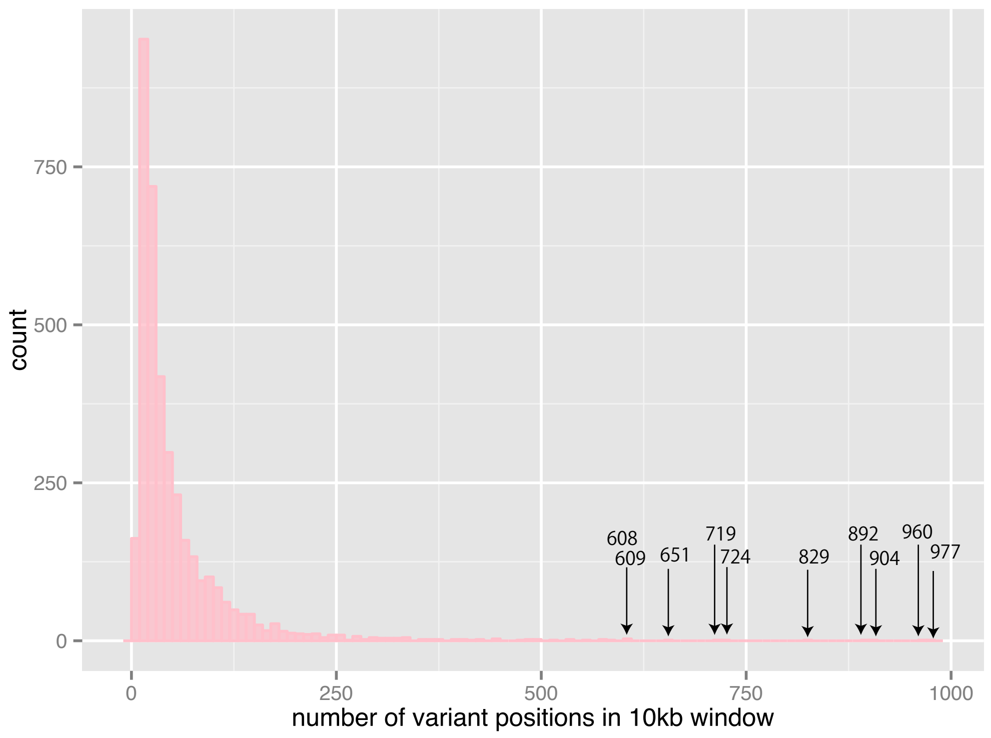

S3 Fig. A) Number of variant positions among 33 studied samples in 10-kb window along the four largest scaffolds/contigs in Japanese and Myanmar *S. stercoralis* nematodes compared with the reference genome. B) A histogram showing SNP number dsitributions in 10-kb window for saffolds larger than 100 kb. Ten largest numbers represeting hotspots are shown with black arrows.
