# Supplementary material for: Genome-Wide Analyses of Individual Strongyloides stercoralis (Nematoda: Rhabditoidea) Provide Insights into Population Structure and Reproductive Life Cycles
Source: PLoS Negl Trop Dis. 2016 Dec 29;10(12):e0005253. doi: 10.1371/journal.pntd.0005253 (PMC5226825; doi:10.1371/journal.pntd.0005253)
Supplement: S4 Fig — Trees were constructed using FastTree and visualised using FigTree. Two haplotypes from a diploid genome are coloured in either red or blue. (PDF) [file pntd.0005253.s008.pdf]

A

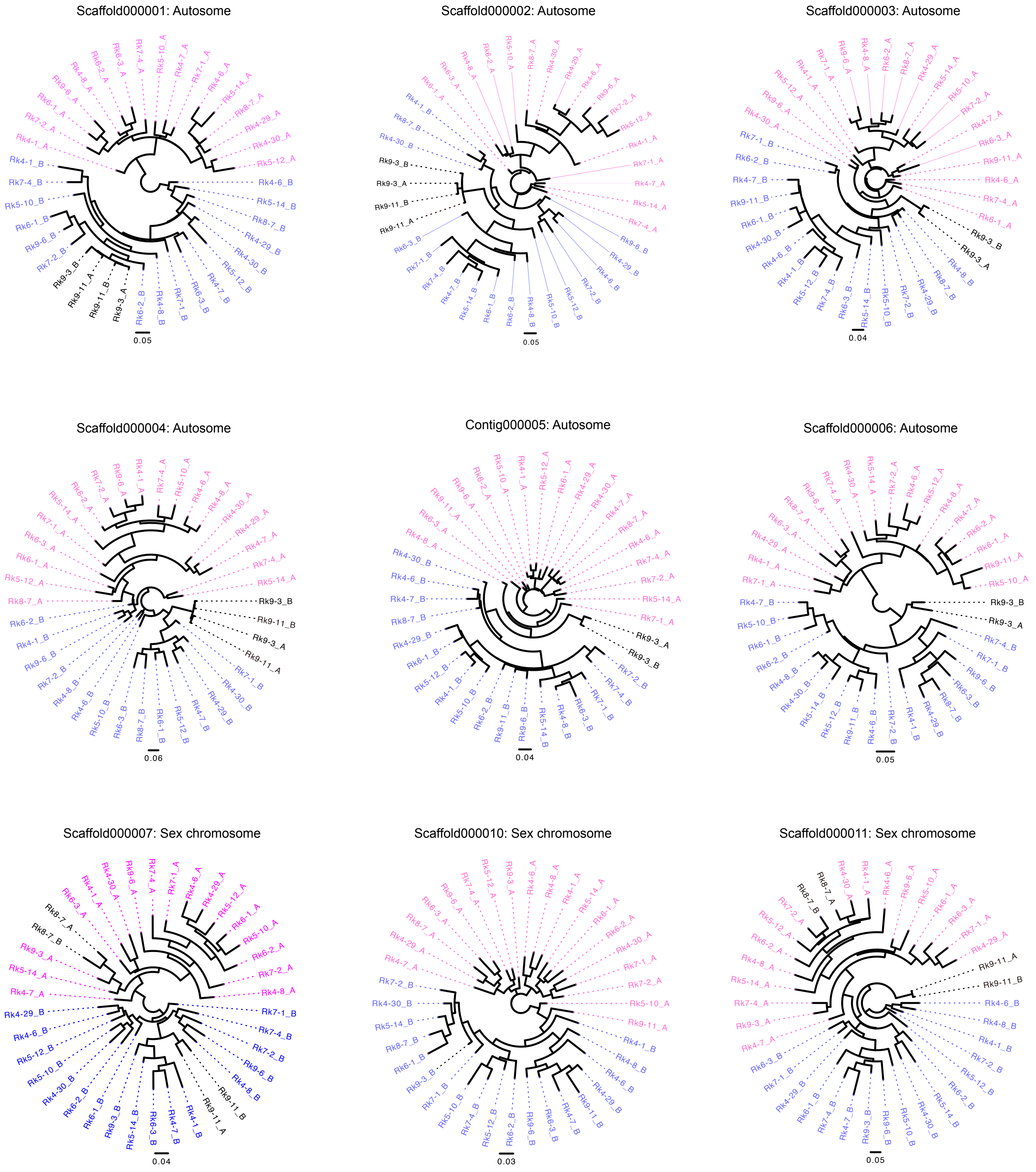

Scaffold000001: Autosome
0.05
Scaffold000002: Autosome
0.05
Scaffold000003: Autosome
0.04
Scaffold000004: Autosome
0.06
Contig000005: Autosome
0.04
Scaffold000006: Autosome
0.05
Scaffold000007: Sex chromosome
0.04
Scaffold000010: Sex chromosome
0.03
Scaffold000011: Sex chromosome
0.05

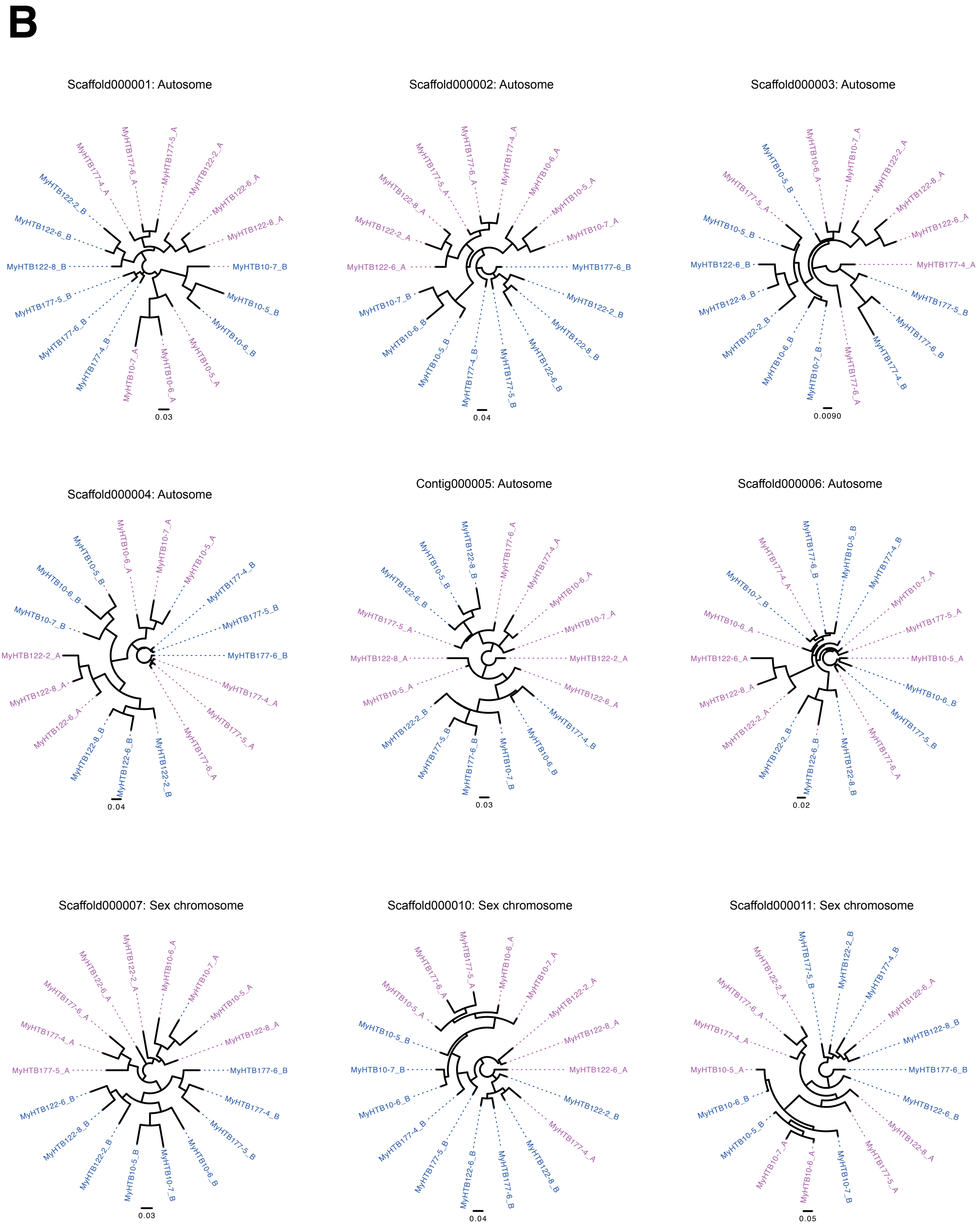

Fig. S4. Maximum likelihood trees of phased haplotype sequences of Scaffold000001, Scaffold000002, Scaffold000003, Scaffold000004, Contig000005, Scaffold000006 (autosomes) and Scaffold000007, Scaffold0000010, Scaffold0000011 (sex chromosomes). Trees were constructed using FastTree and visualised using FigTree. Two haplotypes from a diploid genome are coloured in either red or blue.
