# Supplementary material for: Genome-Wide Analyses of Individual Strongyloides stercoralis (Nematoda: Rhabditoidea) Provide Insights into Population Structure and Reproductive Life Cycles
Source: PLoS Negl Trop Dis. 2016 Dec 29;10(12):e0005253. doi: 10.1371/journal.pntd.0005253 (PMC5226825; doi:10.1371/journal.pntd.0005253)
Supplement: S5 Fig — (PDF) [file pntd.0005253.s009.pdf]

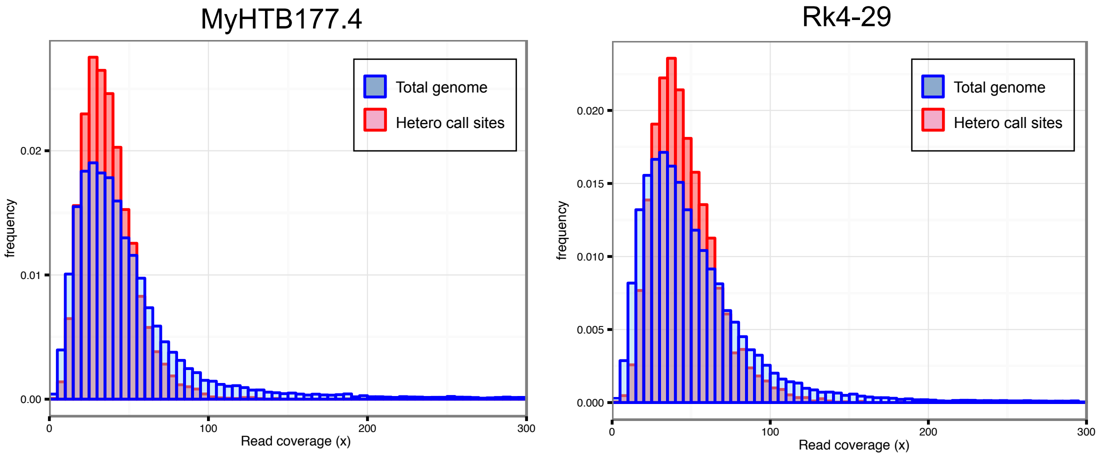

S5 Fig. Comparison of depth of coverage of the whole genome sites and of heterozygous SNP sites, suggesting heterozygous SNP call is not highly affected by depth of reads coverage.
