# Supplementary material for: Genome-Wide Analyses of Individual Strongyloides stercoralis (Nematoda: Rhabditoidea) Provide Insights into Population Structure and Reproductive Life Cycles
Source: PLoS Negl Trop Dis. 2016 Dec 29;10(12):e0005253. doi: 10.1371/journal.pntd.0005253 (PMC5226825; doi:10.1371/journal.pntd.0005253)
Supplement: S6 Fig — Excess of heterozygosity sites in Japanese samples are consistently observed in the scaffolds. Out of 1175 windows, 941 (80.1%) and 205 (17.4%) show excess in Japanese and Myanmar samples, respectively and the difference between the two proportion is significant (Z test; p< 2.6e-174). (PDF) [file pntd.0005253.s010.pdf]

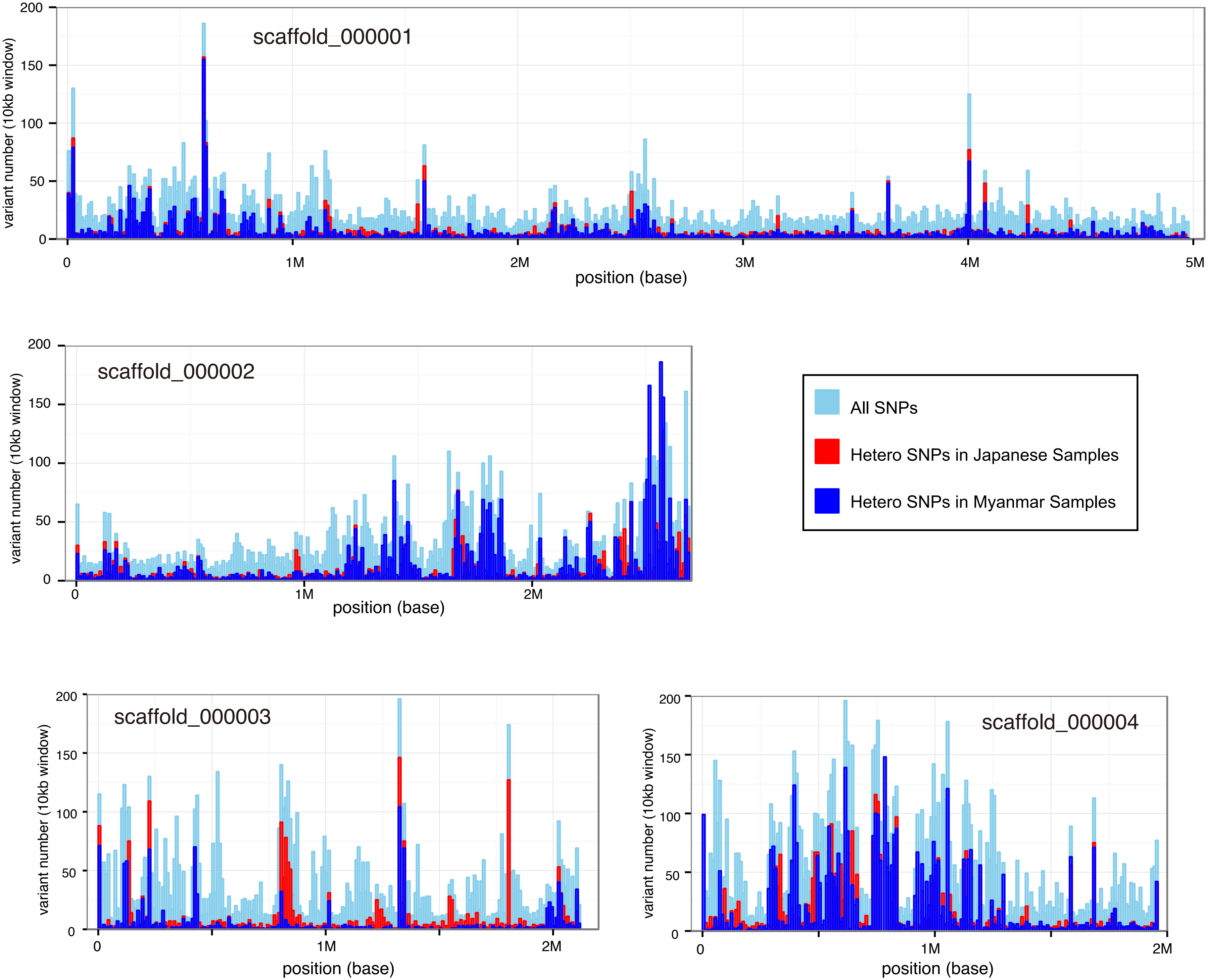

S6 Fig. Distribution of heterozygous SNP sites in Japanese and Myanmar samples along in 10-kb window along the four largest scaffolds/contigs. Excess of heterozygosity sites in Japanese samples are consistently observed in the scaffolds. Out of 1175 windows, 941 (80.1%) and 205 (17.4%) show excess in Japanese and Myanmar samples, respectively and the difference between the two proportion is significant (Z test; $p < 2.6e\text{-}174$).
