# Supplementary material for: Genome-Wide Analyses of Individual Strongyloides stercoralis (Nematoda: Rhabditoidea) Provide Insights into Population Structure and Reproductive Life Cycles
Source: PLoS Negl Trop Dis. 2016 Dec 29;10(12):e0005253. doi: 10.1371/journal.pntd.0005253 (PMC5226825; doi:10.1371/journal.pntd.0005253)
Supplement: S7 Fig — Three areas were shown by different colours and shapes. Broken line represents Y = X. (PDF) [file pntd.0005253.s011.pdf]

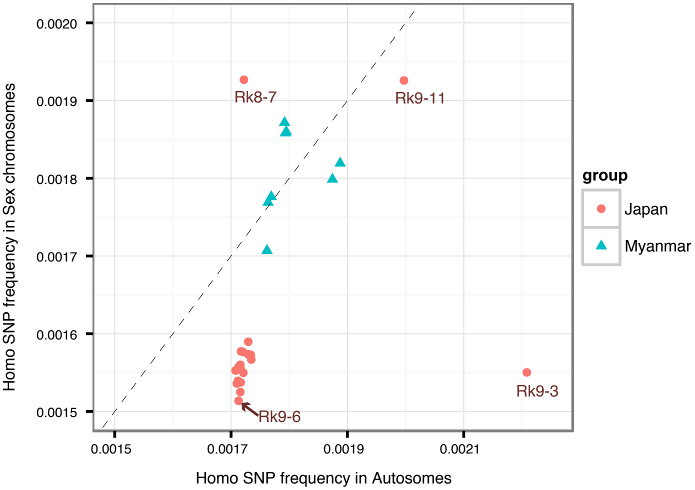

S7 Fig. Frequencies of homozygous SNPs per nucleotide position in autosomes and sex chromosomes. Two areas were shown by different colours and shapes. Broken line represents Y=X.
